# Impact of Body Mass Index and VO2 Max on Symptoms, Physical Activity, and Physical Function in a Multinational Sample of People with HIV

**DOI:** 10.1007/s10461-024-04509-6

**Published:** 2024-09-23

**Authors:** Christine Horvat Davey, Deepesh Duwadi, J. Craig Phillips, Carol Dawson-Rose, Kathleen M. Nokes, Joseph Perazzo, Rebecca Schnall, Penny Orton, Mary Jane Hamilton, Rita Musanti, Kimberly Adams Tufts, Elizabeth Sefcik, Allison R. Webel

**Affiliations:** 1 Frances Payne Bolton SON, Case Western Reserve University, Cleveland, OH 44106 USA; 2 International Nursing Network for HIV Research, San Francisco, CA USA; 3 University of Ottawa, Ottawa, ON Canada; 4 University of California San Francisco, San Francisco, CA USA; 5 School of Professional Studies, City University of New York, New York City, NY USA; 6 University of Cincinnati, Cincinnati, OH USA; 7 Columbia University, New York, NY USA; 8 Durban University of Technology, Greyville, Durban South Africa; 9 Texas A & M University, Corpus Christi, TX USA; 10 Rutgers Cancer Institute of New Jersey, New Brunswick, NJ USA; 11 Old Dominion University, Norfolk, VA USA; 12 University of Washington SON, Seattle, WA USA

**Keywords:** HIV, Symptoms, Physical Activity, 6-minute Walk Test, VO_2_ max

## Abstract

People with HIV (PWH) are at increased risk for metabolic disorders affecting body mass index (BMI), chronic symptoms, and impaired physical function and capacity. Although physical activity improves health and well-being, PWH often do not meet activity recommendations necessary to achieve these benefits. Despite the known impact of symptoms, physical activity, and physical function on health, little is known about the relationships and interactions between these variables and BMI and maximum oxygen consumption during exercise (VO_2_ max) in a multinational population of PWH. We examined the relationship of BMI with PROMIS-29 measures, physical activity, strength, flexibility, and VO_2_ max in a diverse sample of PWH. Additionally, we examined the relationship of VO_2_ max with PROMIS-29 measures. Data from 810 PWH who participated in a cross-sectional study conducted by the International Nursing Network for HIV Research (Study VII) were analyzed. Participants were recruited from 8 sites across the United States, Thailand, and South Africa. BMI was calculated from collected height and weight data. Physical function and symptoms were assessed using the PROMIS-29 measure. Physical activity was assessed using the 7-day Physical Activity Recall. VO_2_ max was calculated using sex at birth, age, BMI and the 6-minute Walk Test. Data were analyzed using descriptive, correlational, and regression statistical analyses. Participants had an average age of 49.1 (± 11.1) years, 44% were female, and the average BMI of the sample group was 27 kg/m2 (± 6.7). Increased BMI was associated with decreased 6-minute Walk Test (β=-2.18, *p* < 0.001), flexibility (β=-0.279, *p* < 0.001), and VO_2_ max (β=-0.598, *p* < 0.001), even after controlling for covariates (age, sex at birth, country, years living with HIV, and antiretroviral therapy status). BMI was not associated with self-reported physical activity. Increased VO_2_ max was associated with increased physical function (β = 0.069, *p* < 0.001), and decreased pain (β=-0.047, *p* < 0.006), even after controlling for covariates (country, years living with HIV, and antiretroviral therapy status). Future research should explore development of effective and sustainable symptom self-management interventions in PWH accounting for the potential impact of BMI and VO_2_ max.

## Introduction

People with HIV (PWH) have an elevated risk for metabolic disorders that impact body mass index (BMI), such as alterations in body composition and increased body fat [1] as well as increased frequency of chronic symptoms [2] and diminished physical function [3] and capacity [4]. BMI in PWH can be impacted by various factors, including treatment with antiretroviral therapy (ART), though the underlying mechanism for weight gain is not conclusive [5]. In addition to weight gain, initiation of ART in PWH can lead to lipodystrophy (fat redistribution), and altered fat metabolism [5, 6]. Some weight gain has been attributed to the return-to-health phenomenon [5]. Risk factors for weight gain in individuals on ART involve low CD4 count (< 200 cells/µL), race, intravenous drug use, and amount of HIV RNA [6]. In addition, black females with HIV experience the most weight gain compared to males, adding to the multifactorial effects of this issue [6]. Some of the ART drug classes such as non-nucleoside reverse transcriptase inhibitors (NNRTIs) are better tolerated with lower incidence of side effects such as nausea, which could result in higher caloric intake [6]. Lower serum leptin levels and off-target effects of certain drugs on the leptin pathway have been suggested, which could alter energy homeostasis and food intake leading to lipodystrophy [7]. In PWH, high BMI is associated with chronic conditions such as cardiovascular disease and diabetes [8], but there is limited literature regarding the association of BMI and symptoms, physical activity, physical function, strength, flexibility, and maximal oxygen consumption (VO_2_ max) in a large multinational sample of PWH.

As HIV has transformed from an acute to a chronic condition [9] and with the era of ART, PWH are living longer and in turn experiencing more symptoms associated with various factors, including the illness, inflammation, and ART [10]. Symptoms in this population are prevalent and can be pervasive; some of the most common symptoms include fatigue experienced by up to 65% [11], sleep disturbances (58%) [12], pain (55%) [11], depressive symptoms (35%) [13], and anxiety (15.5%) [14]. Symptoms can negatively impact PWH; for example, depression is associated with decreased quality of life [15] and sleep disturbance is associated with poor ART adherence [16]. Despite the negative impact of symptoms on PWH, the relationship between symptoms (i.e., fatigue, pain, depressive symptoms, anxiety, participation in social activities, and sleep disturbance) and BMI and VO_2_ max within a demographically diverse sample of PWH has been little studied.

PWH experience accelerated aging [17], which is associated with decreased physical function [18]. Aging virally suppressed PWH on ART demonstrate decreased physical function when compared with age-matched HIV negative counterparts [19]. Poor physical function in PWH is associated with decreased levels of physical activity, yet the relationship between BMI, VO_2_ max, and physical function in this patient population remains unclear.

VO_2_ max refers to the maximum rate of oxygen consumption during exercise. It is generally considered the gold standard measure for cardiorespiratory fitness, including aerobic exercise capacity and physical capacity, and can be measured by performing an incremental VO_2_ max test in a clinical setting or exercise physiology laboratory [20]. VO_2_ max can also be assessed through estimation using the 6-minute Walk Test, the method used in this study. VO_2_ max decreases with age among healthy adults, but a more substantial decline is found in PWH [21]. Exercise is associated with increased VO_2_ max in PWH [22], though limited evidence exists regarding the relationship between VO_2_ max and symptoms in a diverse group of PWH.

BMI and VO_2_ max can serve as indicators of health in PWH, and in turn, evaluation of these factors in relation to health outcomes (i.e., symptoms, physical function) is important. The Symptom Management Theory (SMT) guided this study, as it provides a comprehensive framework for understanding the complex interactions between BMI and VO_2_ max with individual characteristics and outcomes, including symptoms and physical function [23].

PWH experience a myriad of symptoms and earlier physical function impairments, thus understanding modifiable factors (i.e., BMI, VO_2_ max) is vital to developing strategies to manage and mitigate symptoms and optimize physical function. In turn, we explored the impact of BMI on symptoms, physical function (PROMIS-29 measure), physical activity, strength, flexibility, and VO_2_ max, as well as the impact of VO_2_ max on symptoms (PROMIS-29 measure) in PWH.

## Methods

### Study Design

We conducted a secondary analysis of the International Nursing Network for HIV Research Study VII data, a multisite, cross-sectional study to examine cardiorespiratory fitness and physical activity by age and sex, as well as describe the association between cardiorespiratory fitness and physical activity in PWH [24]. The outcome of the study was cardiorespiratory fitness and the primary predictor variables were age, sex, and physical activity [24]. Demographic, anthropomorphic, and clinical variables were used to describe the sample or to examine as possible modifiers [24]. Local Institutional Review Board (IRB) approval was obtained from each participating site, with primary IRB approval from University Hospital’s Cleveland Medical Center. All site Principal Investigators contributed to the study design, were trained, and certified in each study procedure, and obtained local IRB approval prior to any data collection [24].

### Sample

In this study, 810 participants who were included and completed it were recruited from eight sites across the United States, Thailand, and South Africa. The sites in the United States included Cleveland, Ohio; Newark, New Jersey; New York City, New York; Norfolk, Virginia; Corpus Christi, Texas; and San Francisco, California. Bangkok and Durban were the sites in Thailand and South Africa, respectively. Inclusion criteria for the study included ≥ 18 years of age and a verified positive HIV test (HIV + ELISA with confirmatory polymerase chain reaction or Western blot). Exclusion criteria for this study included use of an assistive device (i.e., cane, wheelchair) during physical activity, self-report medical contraindication for exercise testing and training per the American Heart Association criteria [25], and/or on a site-by-site basis, the inability to communicate in the official language of the country, due to feasibility [24].

### Procedures

Written, informed consent was obtained prior to any study procedures from all participants. All study procedures occurred between January 2016 and September 2019. Demographic data, symptom assessment, physical function, BMI, and physical activity characteristics were collected. All data collected was stored in a central Research Electronic Data Capture (REDCap) database stored at the coordinating site. In compliance with local standards, incentives (USD $5 - $50) were provided to participants that completed study procedures.

### Measures

Sociodemographic information was obtained from each participant, while medical characteristics such as duration of HIV infection and current HIV viral load were obtained by medical chart abstraction. The study staff measured participants’ height, weight, and waist and hip circumferences in triplicate, rounded to the nearest centimeter. BMI was calculated from collected height and weight data.

### Symptom Experience and Physical Function

Symptom experience was assessed using the Patient-Reported Outcomes Measurement Information System (PROMIS-29), which measures physical function, fatigue, pain, depressive symptoms, anxiety, participation in social activities, and sleep disturbance. In PWH, the PROMIS-29 is a reliable and valid instrument, with a Cronbach’s α > 0.7 [10]. A lower PROMIS-29 score for fatigue, pain, depressive symptoms, anxiety, participation in social activities, and sleep disturbance indicates the participant experienced less of the symptom, while a lower PROMIS-29 physical function score indicates increased physical function [10]. Questionnaires were provided in Spanish as needed to participants if the study site had staff with the capability of administering the Spanish version.

### Physical Activity

Physical activity was assessed using the 7-day Physical Activity Recall (PAR), a semi-structured interview that approximates the time spent in light, moderate, and vigorous physical activity for the past 7 days in bouts of more than 10 min [26]. The 7-day PAR is considered a valid and reliable measure [26]. There is convergent validity between self-reported physical activity in the 7-day PAR and that of accelerometry (*r* = 0.52) [26]. Participants were asked to recall their activity over the past 7 days after trained study staff defined light, moderate, and vigorous physical activity to the participant. Trained study staff assisted participants through the recall process to assess duration and intensity of the physical activities.

### 6-minute Walk Test

Aerobic capacity and endurance was assessed using the validated 6-minute Walk Test in accordance with the American Thoracic Society guidelines [27, 28]. The 6-minute Walk Test involves participants walking as far as possible, back and forth on a flat, 30-meter-long premeasured path for 6 min. The distance walked was approximated to the closest meter.

### Strength

Hand grip strength is a validated marker of upper muscle strength capacity [29]. Hand grip strength was measured by a dynamometer, and measures were completed in duplicate for each hand [29]. The final hand grip score was attained by averaging the two scores for each hand.

### Flexibility

Flexibility was assessed with the Back Scratch Test, which measures how close in proximity the hands can be brought together behind one’s back while standing. Participants initiated the test by assuming an upright position, positioning one arm/hand on their lower back, and guiding it upward along the spine toward the head [30]. Simultaneously, the opposite arm/hand was positioned behind the neck, tracing a downward path along the spine [30]. The objective was to bring the long finger of each hand into proximity or overlap as much as possible. Measurement of the gap between the fingertips of the long fingers were obtained, with positive values indicating overlap and negative values signifying a lack of meeting between the fingers [30]. The results were recorded to the nearest half inch and performed in duplicate with the best score of both trials being recorded.

### VO2 Max

VO_2_ max (V- volume; O_2_ – oxygen; max – maximum, normalized per kilogram of body mass) is the maximum rate of oxygen consumption possible during physical exertion [31, 32]. VO_2_ max is considered the gold standard measure of aerobic exercise capacity, as well as an individual’s physical capacity [20]. A VO_2_ max test measures the amount of oxygen inhaled and exhaled, which gives accurate information on how well the body is utilizing oxygen. This is usually done on a treadmill or a bike at incremental intensity. Another alternative to measuring VO_2_ max involves data from the 6-minute Walk Test. Submaximal VO_2_ testing is cost-effective, safer, and does not require highly trained personnel, which makes it a preferred choice to maximal testing [31]. The 6-minute Walk Test can be used as a simple tool to evaluate VO_2_ max in low resource settings [31, 32]. We utilized the following reference equation to assess objectively measured VO_2_ max, based on the 6-minute Walk Test, sex at birth, age, and BMI: VO_2_ max (mL/kg/min) = 59.44 − 3.83*sex (1—men; 2—women) − 0.56*age (years) − 0.48*BMI (kg/m2) + 0.04*6-minute Walk Test (m) (*R* = 0.85, R2 = 72.3%, SEE = 3.99 mL/kg/min, *p* < 0.001) [31, 32]. An average sedentary male (age 35 to 54 years of age) attains a VO_2_ max of approximately 35 to 39 mL/kg/min while an average sedentary female (age 35 to 54 years of age) attains a VO_2_ max of approximately 27 to 30 mL/kg/min [33]. Elite athletes can attain VO_2_ maxes of approximately 85 mL/kg/min [34].

### Statistical Analyses

Statistical analyses were performed in IBM SPSS Statistics (Version 28). Data were cleaned and descriptive statistics were calculated. Demographic and clinical characteristics of the participants were summarized as percentages and standard deviations. We examined demographic and clinical characteristics by sex at birth using two-sample *t*-tests and Chi square/Fisher’s exact tests. We examined symptoms, physical function, physical activity, aerobic capacity and endurance, strength, flexibility, and VO_2_ max by sex at birth using two-sample *t*-tests. Based on their distribution, continuous variables were summarized by mean and standard deviation or median and interquartile range. We examined bivariate relationships of BMI with symptoms, physical function, physical activity, aerobic capacity and endurance, strength, flexibility, and VO_2_ max. In the first model, the goal was to evaluate the explanatory contributions of BMI to symptoms, physical function, aerobic capacity and endurance, physical activity, strength, flexibility, and VO_2_ max. The second model is adjusted for covariates: sex at birth and age (independent variable of BMI) or sex at birth, age, and BMI (independent variable of VO_2_ max). Furthermore, we examined bivariate relationships of VO_2_ max with symptoms and physical function (PROMIS-29 measure). In the first model, the goal was to evaluate the explanatory contributions of VO_2_ max to symptoms and physical function (PROMIS-29 measure). Multivariable linear models were conducted to examine the relationships between (1) BMI and symptoms and physical function (PROMIS-29 measure), physical activity, aerobic capacity and endurance, strength, flexibility, and VO_2_ max; along with (2) VO_2_ max and symptoms and physical function (PROMIS-29 measure).

## Results

Of the 810 people that completed the study, the mean age of participants was 49.1 years (± 11.1), 44% were female (*n* = 357), mean years diagnosed with HIV was 18.6 years (± 9.5), mean BMI was 27.0 kg/m^2^ (± 6.7) and mean estimated VO_2_ max was 29.9 ml/kg/min (± 9.9). Out of 810 participants enrolled, 609 participants were enrolled from the United States, 101 from Thailand, and 100 from South Africa. 52.2% of the participants from the United States identified as White, 99% from Thailand identified as Thai, and 99% from South Africa identified as Black. 93% of the total populations were taking ART medications, and 58.4% had comorbidities. Years with HIV diagnosis and HIV viral load suppression were significantly different between female and male participants (t(753) = 2.62, *p* < 0.05) and (X^2^(1) = 6.31, *p* < 0.05) respectively, with males having HIV longer and a higher proportion of viral suppression. BMI between females and males were also statistically different (t(654) = -3.93, *p* < 0.001) as well as estimated VO_2_ max (t(699) = 8.47, *p* < 0.001), with females having a higher BMI and lower VO_2_ max. There was a statistically significant difference in the proportion of males with comorbidities compared to females (X^2^(1) = 8.59, *p* = 0.003). However, hip/waist ratio and proportion of people on ART did not significantly differ by sex at birth. Demographic and medical characteristics of the sample are presented in Table 1.

**Table 1:** Demographic and Medical Characteristics Stratified by Sex at Birth

	Total	Female	Male	Difference		
	*N* (%) or Mean (SD)	*N* (%) or Mean (SD)	*N* (%) or Mean (SD)	Test df	Values (t-test or chi-square)	*p*-value
Age (years)	49.1 (11.1)	48.7 (11.4)	49.3 (11.9)	803	0.597	0.551^a^
Sex at birth	**-------**	357 (44)	453 (56)			**-------**
Country				2	72.1	< 0.001
United States Thailand South Africa	609 (75.2) 101 (12.5) 100 (12.3)	225 (63) 50 (14.1) 82 (22.9)	384 (84.7) 51 (11.4) 18 (3.9)	1 1 1	50.6 1.38 66.6	
Marital status Married Widowed Single Separated Divorced Other	808 163 (20.17) 64 (7.92) 446 (55.20) 46 (5.69) 79 (9.78) 10 (1.24)	356 74 (20.79) 47 (13.20) 160 (44.94) 30 (8.43) 41 (11.52) 4 (1.12)	452 89 (19.69) 17 (3.76) 286 (63.27) 16 (3.54) 38 (8.40) 6 (1.33)	5	45.04	< 0.001*^b^
Highest education 11th grade or less High school or GED Technical/vocational College/university Master’s degree or higher	806 193 (23.9) 243 (30.1) 281 (34.9) 64 (7.9) 25 (3.1)	356 113 (31.7) 114 (32) 110 (30.9) 14 (3.9) 5 (1.4)	450 80 (17.8) 129 (28.7) 171 (38.0) 50 (11.1) 20 (4.4)	4	38.6	< 0.001*^b^
Has health insurance	714 (88.1)	324 (86.1)	390 (90.8)	1	4.15	0.041*^b^
Working status Currently working Laid off Unemployed Retired On disability Others	809 230 (28.4) 12 (1.5) 155 (19.2) 58 (7.2) 274 (33.9) 80 (9.9)	356 88 (24.7) 6 (1.7) 77 (21.6) 19 (5.3) 114 (32) 52 (14.7)	453 142 (31.3) 6 (1.3) 78 (17.2) 39 (8.6) 160 (35.3) 28 (6.1)	5	23.2	< 0.001*^b^
Permanent housing	603 (74.6)	267 (75)	336 (74.3)	1	0.046	0.830^b^
Suppressed viral load	649 (88)	301 (91.8)	348 (86)	1	6.312	0.012*^b^
Years with HIV	18.6 (9.5)	17.6 (8.9)	19.4 (9.9)	753	2.627	0.010*^a^
People on ART	724 (93)	328 (45.3)	396 (54.7)	1	0.581	0.446^b^
Comorbidities	447 (58.4)	182 (40.7)	265 (59.3)	1	8.597	0.003*^b^
Waist/hip ratio	0.84 (0.16)	0.83 (0.15)	0.85 (0.16)	786	1.86	0.063^a^
Body mass index (kg/m^2^)	27.0 (6.7)	28.0 (7.4)	26.1 (5.9)	654	-3.932	< 0.001*^a^

Note: *Significant, p-value ≤ 0.05. ^a^*p*-value from two-sample *t*-test; ^b^*p-*value from Chi square/Fisher’s exact tests

### **Symptoms**,** Physical Function**,** Physical Activity**,** Aerobic Capacity and Endurance**,** Strength**,** Flexibility**,** and VO2 Max Stratified by Sex**

We examined differences in symptoms (PROMIS-29 outcomes, i.e., physical function, fatigue, pain, depressive symptoms, participation in social activities, sleep disturbances) as reported by sex at birth (Table 2). Females experienced more pain compared to males (t(706) = -3.13, *p* = 0.002). Males reported more minutes of light (t(763) = 2.29) and moderate (t(763) = 3.0) physical activity per week on the 7-day PAR than females (*p* < 0.05). Compared to females, males had better aerobic capacity and endurance assessed by the 6-minute Walk Test (t(798) = 6.49, *p* < 0.001), increased strength assessed by hand grip strength (t(791) = 13.02, *p* < 0.001), and increased aerobic exercise capacity assessed by VO_2_ max (t(699) = 8.47, *p* < 0.001).

**Table 2:** Symptoms, Physical Activity, Physical Function, Strength, Flexibility, and VO_2_ Max Stratified by Sex

	Total		Female		Male		Difference		
	*N*	Mean (SD)	*N*	Mean (SD)	*N*	Mean (SD)	t-value	df	*p*-value
Physical function	809	17.7 (3.3)	357	17.4 (3.5)	452	18.0 (3.1)	2.66	719	0.070
Fatigue	809	8.8 (4.2)	356	9.1 (4.3)	453	8.6(4.1)	-1.59	807	0.114
Pain	809	8.0 (4.5)	357	8.6 (4.8)	452	7.6 (4.2)	-3.13	706	0.002*
Depressive symptoms	810	7.5 (4.0)	357	7.4 (4.0)	453	7.7 (4.0)	1.10	808	0.269
Anxiety	787	8.2 (4.1)	345	8.1 (4.2)	442	8.3 (4.1)	0.35	785	0.720
Participation in social activities	809	16.2 (4.0)	356	16.2 (4.1)	453	16.2 (4.0)	0.26	807	0.799
Sleep disturbance	809	10.7 (4.1)	356	10.6 (4.0)	453	10.8 (4.2)	0.73	807	0.464
7-day PAR (minutes/week) ^a^ Light Moderate Vigorous	810	90 (3.6–225) 50 (0-180) 0 (0–80)	357	75 (2.8–210) 45 (0-150) 0 (0–0)	453	105 (4.4) 55 (0-210) 0 (0–60)	2.29 3.01 -0.93	682 713 764	0.023* 0.003* 0.354
6-minute Walk Test (total meters)	800	402 (95)	354	378 (98)	446	421 (88)	6.49	798	< 0.001*
Hand grip strength (kilograms)	808	47.7 (24.6)	356	36.9 (17.8)	452	56.3 (25.8)	13.02	791	< 0.001*
Flexibility (back scratch in inches)	797	-3.5 (9.9)	352	-3.5 (9.2)	445	-3.4 (10.5)	0.11	786	0.910
VO_2_ max (mL/kg/min)	810	29.9 (9.9)	357	26.6 (10.3)	453	32.5 (8.8)	8.47	699	< 0.001*

Note: *Significant, *p*-value ≤ 0.05. Raw scores for each PROMIS variable are used. ^a^ Median and interquartile range. VO_2_ Max – maximum rate of oxygen consumption. PAR – physical activity recall

### BMI and Symptoms, Physical Function, Physical Activity, Aerobic Capacity and Endurance, Strength, Flexibility, and VO _2_ Max

We examined the unadjusted and adjusted associations between BMI with symptoms and physical function (PROMIS-29 measure), physical activity, aerobic capacity and endurance, strength, flexibility and VO_2_ max through linear regression models (Table 3). The unadjusted association between BMI and physical function (F(1,786) = 15.6), participation in social activities (F(1,785) = 6.3), sleep disturbance (F(1,785) = 6.4), 6-minute Walk Test (aerobic capacity and endurance) (F(1,786) = 18.5); flexibility (F(1,776) = 48.7), and VO_2_ max (F(1,786) = 230) were statistically significant (all *p* < 0.05). Increased BMI is associated with decreased physical function, participation in social activities, aerobic capacity and endurance, flexibility, and VO_2_ max, as well as increased sleep disturbance. The associations between BMI and physical function (F(6,728) = 18.5), participation in social activities (F(6,727) = 14.8), and sleep disturbance (F(6,727) = 11.6), were no longer significant after adjusting for covariates; age, sex at birth, years living with HIV, country, and ART status. The associations between BMI and 6-minute Walk Test (aerobic capacity and endurance) (F(6,720) = 10.3), flexibility (F(6,719) = 23.4), and VO_2_ max (F(4,734) = 86.7) remained statistically significant after adjusting for covariates; age, sex at birth, years living with HIV, country, and ART status (all *p* < 0.01). Additionally, after adjusting for covariates, age, sex at birth, years living with HIV, country, and ART status, the association between BMI and strength (hand grip strength) was statistically significant (*p* < 0.015), with increased BMI associated with increased strength (F(6,726) = 54.0).

**Table 3:** Contribution of BMI to Symptoms, Physical Activity, Physical Function, Strength, Flexibility, and VO_2_ Max

Model	Predictor Variable	Dependent Variables	B	SE	β	*R* ^2^	F	*p*-value
Model 1 (unadjusted)	BMI	Physical function Fatigue Pain Depressive symptoms	-0.069 0.032 0.041 0.011	0.018 0.023 0.024 0.022	-0.139 0.051 0.062 0.017	0.019 0.003 0.004 0.00	15.6 2.0 2.9 0.2	< 0.001* 0.156 0.084 0.626
		Anxiety Participation in SA Sleep disturbance PA self-efficacy 7-day PAR (minutes/week) 6-minute Walk Test (total m) Hand grip strength (kg) Flexibility (back scratch) (in) VO_2_ max (mL/kg/min)	0.025 -0.054 0.056 0.001 0.000 -2.183 0.239 -0.364 -0.689	0.022 0.021 0.022 0.001 0.002 0.508 0.128 0.052 0.045	0.040 -0.089 0.090 0.034 0.006 0.023 0.066 -0.243 -0.476	0.002 0.008 0.008 0.001 0.000 -0.152 0.004 0.059 0.226	1.3 6.3 6.4 0.9 0.1 18.5 3.5 48.7 230.0	0.262 0.012* 0.011* 0.342 0.872 < 0.001* 0.063 < 0.001* < 0.001*
Model 2 (adjusted)	BMI	Physical function Fatigue Pain Depressive symptoms	-0.021 -0.015 -0.011 -0.025	0.018 0.024 0.025 0.022	-0.045 -0.024 -0.017 -0.043	0.132 0.068 0.083 0.092	18.5 8.9 10.9 12.3	0.231 0.533 0.659 0.256
		Anxiety Participation in SA Sleep disturbance PA self-efficacy 7-day PAR (minutes/week) 6-minute Walk Test (total m) Hand grip strength (kg) Flexibility (back scratch) (in) VO_2_ max (mL/kg/min)	-0.019 -0.002 0.004 0.001 -0.002 -2.18 0.282 -0.279 -0.598	0.023 0.022 0.023 -- 0.002 0.545 0.115 0.053 0.045	-0.032 -0.004 0.007 0.061 -0.030 -0.154 0.081 -0.194 -0.421	0.096 0.109 0.087 0.010 0.073 0.079 0.309 0.164 0.321	12.5 14.8 11.6 1.3 9.6 10.3 54.0 23.4 86.7	0.403 0.921 0.863 0.124 0.440 < 0.001* 0.015* < 0.001* < 0.001*

Note: *Significant, *p*-value ≤ 0.05. B is the unstandardized coefficient regression coefficient. SE standard error. β is the standardized regression coefficient. R^2^ = coefficient of determination shown for each model. Model 1 is unadjusted, model 2 is adjusted for covariates: age, sex at birth, years living with HIV, country, and antiretroviral therapy status, except for VO_2_ max, which is adjusted for only years living with HIV, country, and antiretroviral therapy status as it accounts for birth sex and age in its equation. VO_2_ max – maximum rate of oxygen consumption. PA – physical activity. PAR – physical activity recall. SA – social activities. M – meters. Kg – kilograms. In – inches

### VO2 Max and Symptoms

We examined the unadjusted and adjusted associations between VO_2_ max and symptoms (PROMIS-29 measure) through linear regression models (Table 4). The unadjusted associations between VO_2_ max and physical function, fatigue, pain, participation in social activities, and sleep disturbance were statistically significant (all *p* < 0.05). Increased VO_2_ max was associated with increased physical function (F(1,807) = 69.7) and participation in social activities (F(1,807) = 18.8), as well as decreased fatigue (F(1,807) = 9.7), pain (F(1,807) = 25.6), and sleep disturbances (F(1,807) = 3.7). The VO_2_ max regression equation considers age, sex at birth, BMI, and physical function capability as determined from the 6-minute Walk Test. As a result, we did not run the linear regression model adjusting for covariates of age or sex at birth, but we did adjust for country, years living with HIV, and ART status. The associations between VO_2_ max and fatigue (F(4,752) = 11.2), participation in social activities (F(4,752) = 23.6), and sleep disturbance (F(4,752) = 17.4), were no longer significant after adjusting for covariates, years living with HIV, country, and ART status. The adjusted associations between VO_2_ max and physical function (F(4,752) = 27.3) and pain (F(4,752) = 12.4), remained statistically significant (both *p* < 0.05). Additionally, after adjusting for covariates; years living with HIV, country, and ART status, the association between VO_2_ max and depressive symptoms was statistically significant (*p* = 0.046), with increased VO_2_ max associated with increased depressive symptoms (F(4,753) = 16.1).

**Table 4:** Contribution of VO_2_ Max to Symptoms

Model	Predictor Variable	Dependent Variables	B	SE	β	*R* ^2^	F	*p*-value
Model 1 (unadjusted)	VO_2_ max	Physical function Fatigue Pain Depressive symptoms	0.093 -0.046 -0.079 0.011	0.011 0.015 0.016 0.014	0.282 -0.109 -0.175 0.027	0.080 0.012 0.031 0.001	69.7 9.7 25.0 0.6	< 0.001* 0.002* < 0.001* 0.447
		Anxiety Participation in social activities Sleep disturbance	-0.002 0.061 -0.028	0.015 0.014 0.014	-0.004 0.151 -0.068	0.000 0.023 0.005	0.1 18.8 3.7	0.919 < 0.001* 0.050*
Model 2 (adjusted)	VO_2_ max	Physical function Fatigue Pain Depressive symptoms	0.069 -0.011 -0.047 0.030	0.012 0.016 0.017 0.015	0.213 -0.026 -0.105 0.076	0.127 0.056 0.062 0.079	27.3 11.2 12.4 16.1	< 0.001* 0.505 0.006* 0.046*
		Anxiety Participation in social activities Sleep disturbance	0.025 0.023 0.013	0.016 0.015 0.015	0.060 0.057 0.033	0.081 0.112 0.085	16.1 23.6 17.4	0.117 0.129 0.388

Note: *Significant, p-value ≤ 0.05. B is the unstandardized coefficient regression coefficient. SE standard error. β is the standardized regression coefficient. R^2^ = coefficient of determination shown for each model. Model 1 is unadjusted, model 2 is adjusted for covariates: country, years living with HIV and antiretroviral therapy status. VO_2_ Max – maximum rate of oxygen consumption

## Discussion

In this study, we examined the relationship between BMI with symptoms, physical function, physical activity, aerobic capacity and endurance, strength, flexibility, and VO_2_ max along with the relationship of VO_2_ max with symptoms and physical function in a multinational, multisite sample of PWH. We found that increased BMI is associated with decreased aerobic capacity and endurance (6-minute Walk Test), flexibility, and VO_2_ max, as well as increased strength after adjusting for covariates (i.e., age, sex at birth, years living with HIV, country, and ART status). Body mass can affect one’s aerobic capacity and endurance, a study of 90 obese adults demonstrated a positive relationship in both men and women between fat-free mass and distance walked during the 6-minute Walk Test [35]. Another study of 904 adult men and women between the ages of 67–84 years old found fat mass was significantly and inversely correlated with physical capacity [36]. Our findings are supported by the literature in that increased BMI (obesity) is associated with decreased aerobic capacity and endurance as measured by the 6-minute Walk Test [35, 36]. ART can lead to changes in body composition, including lipodystrophy, and in turn, impact aerobic capacity and endurance in PWH [5]. Together, these studies suggest BMI plays a role in aerobic capacity and endurance and interventions to improve BMI in PWH worldwide are needed.

Our study results demonstrated that increased BMI was associated with decreased flexibility. However, there were inconsistent findings regarding the relationship of BMI and flexibility; for example, in a study of 570 young adults by Gite, Mukkamala, and Parmer (2021), no association was found between BMI and flexibility [37]. Results of this study are possibly attributed to physical inactivity in the sample, as 61% of the participants were classified as inactive, and inactivity can contribute to decrease flexibility [37]. In contrast, in a study of 17,970 Taiwanese adults, BMI was inversely associated with flexibility [38]. A potential explanation for our finding includes the impact of ART on BMI and body composition in PWH. The use of modern ART drugs, including integrase strand transfer inhibitors, is associated with increased weight gain and higher BMI, and might increase the risk of weight-related challenges [5] such as decreased flexibility. Addressing the relationship of BMI with flexibility is important when considering strategies for improvement of total body health in PWH.

As expected, we found increased BMI was associated with decreased VO_2_ max. Our results are consistent with the literature, as VO_2_ max was significantly lower in BMI-discordant twin pairs that had an increased BMI [39]. In this study, higher moderate to vigorous physical activity and steps per day were associated with lower percent body fat [39]. These data highlight the relationship of BMI and VO_2_ max independent of genetic and environmental factors. Moreover, one study found that in 98 Indian medical students, increased BMI was associated with decreased VO_2_ max [40]. Another study in 45 young adults demonstrated increased BMI was associated with decreased VO_2_ max [41]. Potential explanations for these findings include possible changes in cardiovascular function in severely overweight individuals and the decrease in Type I and increase in Type II muscle fibers in obese individuals, which may impact oxygen uptake [40].

Additionally, our findings present that increased BMI is associated with increased strength. In the general population as well as PWH, hand grip strength has been found to be associated with BMI [42]. Olsen et al. (2015) found decreased BMI was associated with decreased hand grip strength in PWH living in Ethiopia [43]. A possible explanation for this finding includes that approximately 50% of participants initiating ART in the Ethiopia study exemplified signs of malnutrition, characterized by low BMI, which independently predicted lower levels of physical capacity [43]. Kitilya et al. (2022) found low BMI, moderate anemia, and low CD4 were associated with lower hand grip strength among 272 individuals with HIV living in Mwanza [44]. Loss of lean body mass and fat mass is associated with HIV-associated wasting syndrome and results from malabsorption of nutrients, reduced food intake, decreased utilization of nutrients and increased energy use [45]. In turn, weight loss in PWH is associated with weakness and muscle fatigue, which can contribute to decreased grip strength [46]. Various factors, including age, sex, and comorbidities can contribute to or diminish hand grip strength, and in turn, these factors should be considered when assessing the relationship between BMI and hand grip strength.

Among PWH, increased VO_2_ max is associated with increased physical function and depressive symptoms, as well as decreased pain after adjusting for covariates (i.e., years living with HIV, country, and ART status). Cardiorespiratory fitness can be expressed as VO_2_ max and is the gold standard measure of overall fitness [47]. We found increased VO_2_ max is associated with increased physical function in PWH. In elderly adults with end stage renal disease, peak VO_2_ (highest amount of oxygen consumed at peak exercise) was modestly associated with several measures of physical function [48]. Results from this study highlight the importance of maintaining and improving VO_2_ max, as it is associated with various determinants of health. Erlandson et al. (2018) found moderate- and high-intensity exercise was associated with improved VO_2_ max as well as physical function in older adults with HIV [49]. An important implication to this finding is that VO_2_ max and physical function are related and can be improved with exercise in PWH. The relationship between VO_2_ max and physical function is understudied and complex, with many variables contributing to VO_2_ max and physical function, including age, BMI, and physical activity [50].

Among this diverse population of PWH, increased VO_2_ max is associated with decreased pain. The literature yields mixed results regarding the relationship between VO_2_ max and pain [51–53]. One study of 75 adults with chronic lower back pain found no significant relationship between VO_2_ max and pain [51]. In contrast, a study of 108 adults with chronic low back pain found VO_2_ max was significantly lower in both men and women with low back pain compared to healthy controls [52]. One plausible explanation for the decreased level of VO_2_ max is the frequency, duration, and intensity of physical activity engaged in by individuals was lower than before the low back pain started compared to healthy controls. Another study of 13,080 adults with self-reported anxiety and low to moderate cardiorespiratory fitness found they had increased risk of low back pain compared to adults with high cardiorespiratory fitness [53]. Anxiety regarding injury from physical activity in individuals with low back pain may lead to avoidance of physical activity [53]. PWH have low levels of physical activity, particularly women with HIV [54]. It is possible that the relationship between VO_2_ max and pain is associated with behaviors that determine VO_2_ max, such as physical activity [55]. In aging adults, exercise in combination with physical modalities can decrease pain [56].

Our study results demonstrate increased VO_2_ max is associated with increased depressive symptoms. Our results present conflicting results with the literature regarding the relationship between VO_2_ max and depressive symptoms. In 6,728 adults in the Aerobics Center Longitudinal Study, increased cardiorespiratory fitness was associated with lower depressive symptoms [57]. Additionally, a systematic review and meta-analysis found low cardiorespiratory fitness is associated with a 47% greater risk for mental health disorders, including depressive symptoms [58]. A plausible explanation for these results include increased cardiorespiratory fitness is related to physical activity, and physical activity may help to mitigate depressive symptoms through various physiological and psychological mechanisms [58]. In our study, we used submaximal VO_2_ testing, which is cost-effective, but use of a different measurement method (i.e., submaximal versus maximal exercise test) could have contributed to the difference in our results compared to the literature regarding the relationship between VO_2_ max and depressive symptoms.

Our study yielded important results regarding the relationship between BMI with symptoms, aerobic capacity and endurance, flexibility, VO_2_ max and strength, as well as the relationship of VO_2_ max with symptoms and physical function in PWH. It is also important to note the dearth of research regarding the relationship of BMI and VO_2_ max in PWH. Future research should further examine the relationship between BMI and VO_2_ max in PWH, as it potentially has important implications for cardiorespiratory fitness and health outcomes in this population. Additionally, future research should investigate the relationship between VO_2_ max and physical function in PWH while accounting for variables including age, BMI, and physical activity that impact VO_2_ max and physical function.

### Limitations

The current study has limitations that should be considered. First, the cross-sectional study design limits our ability to assess causality. Second, the validity of BMI as a measure of body fat can be challenged. BMI is used to assess weight category (i.e., underweight, healthy weight, overweight, and obesity) in a cost-effective and easy manner. Yet, BMI cannot distinguish the proportions of fat and muscle for individuals [59]. Current consensus reviews indicate that BMI serves as an important tool for healthcare screenings and population surveys, but it is limited in its ability to predict accurately chronic disease risk and assessing excess body fat [60]. While BMI can provide guidance for exercise and nutritional counseling, its reliability in predicting individual health risks is questionable [60]. Individuals identified as overweight by BMI may be physically fit by other indicators, necessitating comprehensive evaluation, diagnosis, and monitoring, using both anthropometric and performance metrics to better understand associated risks [60]. Therefore, BMI thresholds need reconsideration across diverse populations based on variations in body structure, age, and ethnicity [60]. Third, our measure of VO_2_ max was calculated based on the 6-minute Walk Test (a submaximal test) and demographic data, not maximal cardiorespiratory stress testing. Both BMI and VO_2_ max measures were selected based on the limited resources of some of the sites, which would not have allowed for more robust measures of these variables. Fourth, physical activity was assessed using the 7-day PAR, a self-report measure. The 7-day PAR is considered a valid and reliable measure, but actigraphy is considered the gold standard for physical activity measurement. Fifth, our sample included PWH who are in routine HIV clinical care, and in turn, our results may not be generalizable to PWH not involved in routine HIV clinical care. Sixth, our study examines symptoms as the outcome variables, and BMI as well as VO_2_ max as the predictor variables, and as such, it is plausible there is a bidirectional relationship between these variables. Seventh, our study did not collect data on specific classes of ART’s that participants were prescribed. Specific classes of ART can impact body composition, in turn this is important to note [5].

## Conclusions

Our findings indicate that BMI and VO_2_ max are significantly associated with various symptoms in PWH spanning three continents. As HIV continues to transition to a chronic disease that is manageable, prioritizing physical function and its association with VO_2_ max is an important area to understand in this population. BMI and VO_2_ max could serve as potential targets for intervention to improve symptom self-management in PWH. Future research should consider the potential impact of BMI and VO_2_ max on symptom experience, physical activity, and physical function to develop effective and sustainable symptom self-management interventions. Such interventions have the potential to not only improve self-management, but could potentially decrease the risk for metabolic disorders.

## Data Availability

The datasets used and/or analyzed during the current study are available from the corresponding author on reasonable request.

## Code Availability

The codes used for analysis of the current study are available from the corresponding author on reasonable request.
