# Correction: Impact of Body Mass Index and VO2 Max on Symptoms, Physical Activity, and Physical Function in a Multinational Sample of People With HIV

**DOI:** 10.1007/s10461-025-04666-2

**Published:** 2025-03-07

**Authors:** Christine Horvat Davey, Deepesh Duwadi, J. Craig Phillips, Carol Dawson-Rose, Kathleen M. Nokes, Joseph Perazzo, Rebecca Schnall, Penny Orton, Mary Jane Hamilton, Rita Musanti, Kimberly Adams Tufts, Elizabeth Sefcik, Allison R. Webel

**Affiliations:** 1 Frances Payne Bolton SON, Case Western Reserve University, Cleveland, OH 44106 USA; 2 International Nursing Network for HIV Research, San Francisco, CA USA; 3 University of Ottawa, Ottawa, ON Canada; 4 University of California San Francisco, San Francisco, CA USA; 5 School of Professional Studies, City University of New York, New York City, NY USA; 6 University of Cincinnati, Cincinnati, OH USA; 7 Columbia University, New York, NY USA; 8 Durban University of Technology, Greyville, Durban South Africa; 9 Texas A&M University, Corpus Christi, TX USA; 10 Rutgers Cancer Institute of New Jersey, New Brunswick, NJ USA; 11 Old Dominion University, Norfolk, VA USA; 12 University of Washington SON, Seattle, WA USA

In this article, the authors Rebecca Schnall, Penny Orton, Mary Jane Hamilton, Rita Musanti, Kimberly Adams Tufts, Elizabeth Sefcik were missing from the author group.

The author Rebecca Schnall is affiliated to “International Nursing Network for HIV Research, San Francisco, CA, USA” and “Columbia University, New York, NY, USA”

The author Penny Orton is affiliated to “International Nursing Network for HIV Research, San Francisco, CA, USA” and “Durban University of Technology, Greyville, Durban, South Africa”

The author Mary Jane Hamilton is affiliated to “International Nursing Network for HIV Research, SanFra ncisco, CA, USA” and “Texas A&M University, Corpus Christi, TX, USA”

The author Rita Musanti is affiliated to “International Nursing Network for HIV Research, San Francisco, CA, USA” and “Rutgers Cancer Institute of New Jersey, New Brunswick, NJ, USA”

The author Kimberly Adams Tufts is affiliated to “International Nursing Network for HIV Research, San Francisco, CA, USA” and “Old Dominion University, Norfolk, VA, USA”

The author Elizabeth Sefcik is affiliated to “International Nursing Network for HIV Research, San Francisco, CA, USA” and “Texas A&M University, Corpus Christi, TX, USA”

The Acknowledgements section was missing from this article and should have read, “This work was supported by the National Institute of Nursing Research under grants R01NR015737 (Schnall) and T32 NR007081 (Dawson-Rose). Its contents are solely the responsibility of the authors and do not necessarily represent the official view of the National Institutes of Health”.

The original article has been corrected.

